# Consumption of Select Dietary Emulsifiers Exacerbates the Development of Spontaneous Intestinal Adenoma

**DOI:** 10.3390/ijms22052602

**Published:** 2021-03-05

**Authors:** Emilie Viennois, Benoit Chassaing

**Affiliations:** 1 INSERM, U1149, Center of Research on Inflammation, Université de Paris, 75018 Paris, France; emilie.viennois@inserm.fr; 2 INSERM U1016, Team “Mucosal Microbiota in Chronic Inflammatory Diseases”, CNRS UMR 8104, Université de Paris, 75014 Paris, France

**Keywords:** emulsifiers, intestinal adenoma, microbiota, inflammation

## Abstract

Inflammation is a well-characterized critical driver of gastrointestinal cancers. Previous findings have shown that intestinal low-grade inflammation can be promoted by the consumption of select dietary emulsifiers, ubiquitous component of processed foods which alter the composition and function of the gut microbiota. Using a model of colitis-associated cancer, we previously reported that consumption of the dietary emulsifiers carboxymethylcellulose or polysorbate-80 exacerbated colonic tumor development. Here, we investigate the impact of dietary emulsifiers consumption on cancer initiation and progression in a genetical model of intestinal adenomas. In APC^min^ mice, we observed that dietary emulsifiers consumption enhanced small-intestine tumor development in a way that appeared to be independent of chronic intestinal inflammation but rather associated with emulsifiers’ impact on the proliferative status of the intestinal epithelium as well as on intestinal microbiota composition in both male and female mice. Overall, our findings further support the hypothesis that emulsifier consumption may be a new modifiable risk factor for colorectal cancer (CRC) and that alterations in host–microbiota interactions can favor gastrointestinal carcinogenesis in individuals with a genetical predisposition to such disorders.

## 1. Introduction

Colorectal cancer (CRC) is the second most common cancer in women and the third in men worldwide (http://globocan.iarc.fr/Pages/fact_sheets_cancer.aspx?cancer=colorectal, accessed on 1 December 2020). Approximately 5% of cases are associated with highly penetrant inherited mutations [1]. The most commonly inherited syndromes increasing colon cancer risk are familial adenomatous polyposis (FAP) and Lynch syndrome. Germ-line mutations in the adenomatous polyposis coli (APC) tumor suppressor gene are responsible for FAP, an autosomal dominantly inherited disease in humans. Patients with FAP develop multiple benign colorectal polyps progressing to colorectal carcinoma. The lifetime risk of colorectal malignancy in patients with FAP is approaching 100% [2]. Sporadic forms of CRC have also been firmly linked to mutations in the APC gene, with up to 75% of sporadic tumors in CRC patients presenting somatic mutations in APC [3,4,5]. APC^min^ (min, multiple intestinal neoplasia) is a nonsense mutation of the murine homologue of the APC gene. Apc^Min/+^ (APC^min^) mice are predisposed to spontaneous intestinal cancer as they develop multiple intestinal neoplasia and are used to simulate human familial adenomatous polyposis and colorectal tumors [6].

While CRC, with its 5% association with inherited mutations, presents one of the largest proportions of familial cases amongst cancers, it is also one of the so-called westernized diseases, with higher incidence rates in North America, Australia, New Zealand, and Europe (>40 cases per 100,000 people) and lower incidence rates in rural Africa (<5 cases per 100,000 people). Studies on migrant populations provide compelling evidence that environmental factors, rather than genetic predisposition, play a central role in the development of colorectal cancer. Such influence of the environment is for example illustrated by studies of Japanese immigrants to Hawaii who, within one generation, suffered a change in colon cancer incidence from the low rate of the Japanese native population to the high rate of Hawaiian natives [7]. Hence, it is now clear that the origin of CRC is multifactorial, with genetic, molecular, inflammatory, and environmental risk factors. The gut microbiota has also been recognized as an important contributor to CRC initiation and development [8], for example, through *Fusobacterium* [9] and colibactin-producing *Escherichia coli* strains [10,11].

In our attempt to identify environmental factors that can detrimentally impact the intestinal microbiota, we have previously shown that select dietary emulsifiers, i.e., carboxymethylcellulose (CMC) and polysorbate 80 (P80), can alter the microbiota in models of intestinal inflammation and metabolic syndrome [12,13]. Emulsifiers are detergent-like molecules that are incorporated into most processed foods to improve texture and stability, and we observed that these compounds impair mucus–bacterial interactions in a way that induces intestinal inflammation [12]. CMC was previously described to promote overgrowth and small-intestine inflammation in genetically susceptible mice [14], while P80 is able to increase bacterial translocation across epithelia in vitro [15,16]. These two emulsifiers are indigestible and mainly excreted in the feces [17,18,19,20], and both promote microbiota encroachment and an increase of microbiota pro-inflammatory potential in a way that is associated with increased intestinal inflammation [12]. In addition, we also previously reported that consumption of dietary emulsifiers results in an altered gut microbiota composition, with higher proinflammatory potential that creates an intestinal microinflammation sufficient to drive the development of colonic tumors in a mice model (azoxymethane—dextran sulfate sodium (AOM–DSS) of colitis-associated cancer (CAC). Such effects were associated with alterations of the proliferation/apoptosis balance resulting in an increased cell turnover. The microinflammation induced by the emulsifiers was associated with an increased cell turnover that created favorable conditions for exacerbated tumorigenesis in emulsifier-treated mice [21].

In CRC, complex interrelationships with the gut microbiome, inflammation, genetics, and other environmental factors are evident. An altered microbiota can play a role in promoting CAC, not only through the induction of inflammation, but also through the production of toxins that create a favorable niche for tumor cells [22]. Treatment of mice with antibiotics confers some degree of protection against CAC, supporting the idea of a critical role played by the gut microbiota in tumorigenesis [23]. Moreover, azoxymethane (AOM)-treated germ-free IL10^−/−^ mice failed to develop colitis and colorectal tumors, indicating that the presence of colitogenic bacteria is a prerequisite for the development of CAC [24]. In the present study, we hypothesized that dietary emulsifier consumption could aggravate initiation and development of genetically driven CRC. To test this hypothesis, APC^min^ mice, mutated for the tumor suppressor gene APC and prone to develop intestinal adenomas, were subjected to chronic exposure of two select emulsifiers, CMC or P80, and the effects on tumor development, epithelium proliferative status, and microbiota composition were investigated. We report here that CMC- and P80-treated APC^min^ mice developed more tumors, which also were of larger size. The combination of emulsifier exposure with genetic predisposition exacerbated alterations in microbiota composition, reinforcing the concept that environment and genetic participate together in gastrointestinal carcinogenesis, with a central role played by the intestinal microbiota.

## 2. Results

### 2.1. Emulsifiers Increase Polyp Development in APC^min^ Mice

**Figure 1:**
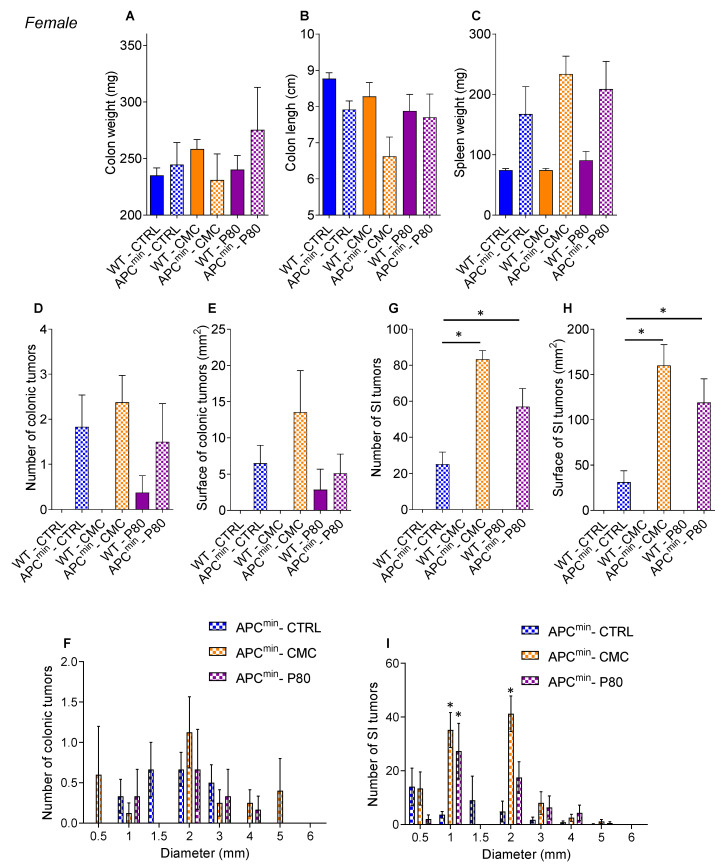
Dietary emulsifier consumption promotes intestinal tumors in adenomatous polyposis coli (APC)^min^ female mice. Seven-week-old wild-type (WT) and APC^min^ female mice were exposed to drinking water containing carboxymethylcellulose (CMC) or polysorbate 80 (P80) (1.0%) for 15 weeks. (**A**) Colon weights, (**B**) colon lengths, (**C**) spleen weights, (**D**) number of colonic tumors, (**E**) total colonic tumor surface determined with an ocular micrometer-fitted dissecting microscope, (**F**) number of colonic tumors based on their size, (**G**) number of small-intestine tumors, (**H**) total small-intestine tumor surface determined with an ocular micrometer-fitted dissecting microscope, (**I**) number of small-intestine tumors based on their size. Data are the means +/− S.E.M. (*n* = 13 for the WT–CTRL group, 6 for the APCmin–CTRL group, 8 for the WT–CMC group, 8 for the APC^min^–CMC group, 8 for the WT–P80 group and 5 for the APC^min^–P80 group). Significance was determined using one-way group ANOVA with a Bonferroni test (* indicates statistical significance).

**Figure 2:**
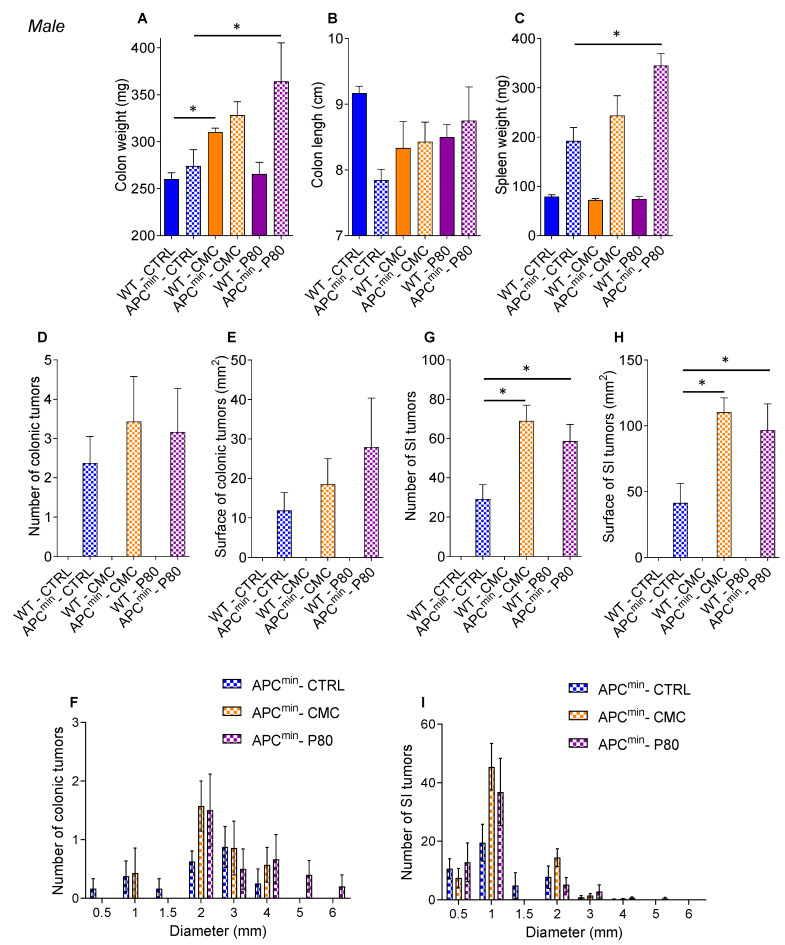
Dietary emulsifier consumption promotes intestinal tumors in APC^min^ male mice. Seven-week-old WT and APC^min^ male mice were exposed to drinking water containing CMC or P80 (1.0%) for 15 weeks. (**A**) Colon weights, (**B**) colon lengths, (**C**) spleen weights, (**D**) number of colonic tumors, (**E**) total colonic tumor surface determined with an ocular micrometer-fitted dissecting microscope, (**F**) number of colonic tumors based on their size, (**G**) number of small-intestine tumors, (**H**) total small-intestine tumor surface determined with an ocular micrometer-fitted dissecting microscope, (**I**) number of small-intestine tumors based on their size. Data are the means +/− S.E.M. (*n* = 6 for the WT–CTRL group, 6 for the APC^min^–CTRL group, 5 for the WT–CMC group, 7 for the APC^min^–CMC group, 7 for the WT–P80 group and 6 for the APC^min^–P80 group). Significance was determined using one-way group ANOVA with a Bonferroni test (* indicates statistical significance).

Seven-week-old female and male wild-type (WT) and APC^min^ mice were treated with either CMC or P80 diluted in drinking water (1.0% *w*/*v*, Figure S1) for 15 weeks, as previously reported [12,21,25]. In order to account for any potential gender effect, females and males were analyzed separately. While P80 exposure tended to increase body weight, in accordance with our previous finding that P80 consumption led to metabolic deregulations, there was no statistically significant difference in body weight between the various groups along the course of the experiment (Figure S2). Analysis of macroscopic parameters of inflammation demonstrated a limited impact of emulsifier consumption on the inflammation level in both WT and APC^min^ mice. More specifically, in males, but not in females, consumption of CMC resulted in an increased colon weight in WT mice compared to the water-administered control mice (Figure 1A–C and Figure 2A–C). Moreover, APC^min^ male mice exposed to P80 showed a significant increase in their colon and spleen weight compared to water-treated mice (Figure 2A–C), which are both features of low-grade inflammation.

Importantly, in both female and male APC^min^ mice, who spontaneously develop adenomas in the gastrointestinal tract, CMC and P80 were sufficient to significantly increase the number and the total surface of polyps in the small intestine compared to the water-treated control group (Figure 1D–H and Figure 2D–H). Analysis of polyp size distribution showed an increased number of 1 and 2 mm-diameter polyps in the small intestine of CMC- and P80-treated female and male APC^min^ mice (Figure 1F–I and Figure 2F–I), while no effect was observed in the colon (Figure 1D,E and Figure 2D,E). Hence, these results importantly indicate that consumption of select dietary emulsifiers can accelerate the formation of intestinal adenomas in genetically predisposed hosts, despite a limited impact on intestinal inflammation.

### 2.2. Emulsifier Consumption Increases Small-Intestine and Colonic Crypt Cells Proliferation in WT and APC^min^ Mice

**Figure 3:**
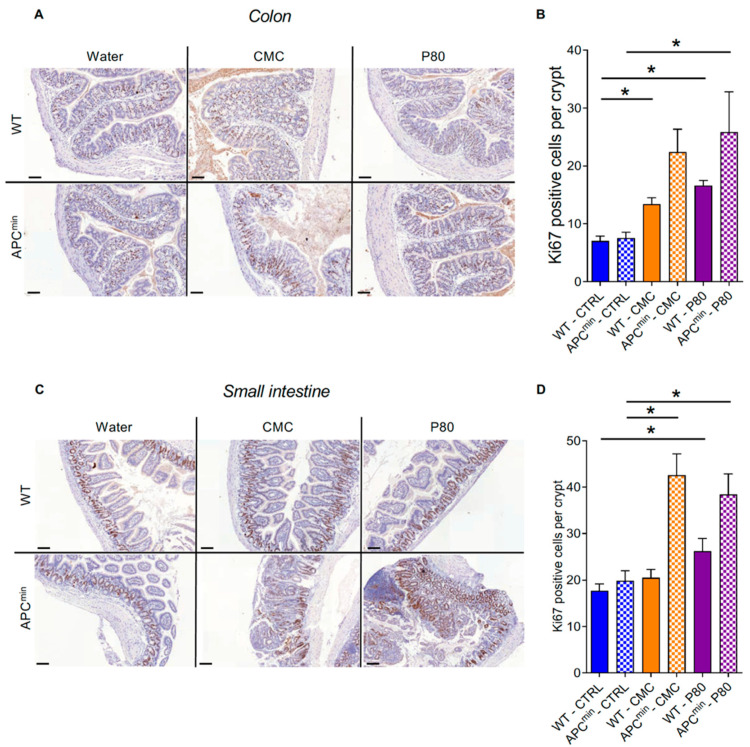
Dietary emulsifier consumption alters epithelial cell proliferation in WT and APC^min^ mice. Seven-week-old WT and APC^min^ female mice were exposed to drinking water containing CMC or P80 (1.0%) for 15 weeks. Epithelial cell proliferation was analyzed by immunohistochemistry using the proliferation marker Ki67 in colonic (**A**,**B**) and small-intestine (**C**,**D**) tissue sections. (**A**) Representative images of Ki67 staining in colonic tissue sections. Scale bar, 100 µm. (**B**) Ki67-positive cells were counted and averaged per crypt in colonic tissue sections. (**C**) Representative images of Ki67 staining in small-intestine tissue sections. Scale bar, 100 µm. (**D**) Ki67-positive cells were counted and averaged per crypt in small-intestine tissue sections. Data are the means +/− S.E.M. (*n* = 11 for the WT–CTRL group, 4 for the APC^min^–CTRL group, 5 for the WT–CMC group, 5 for the APC^min^–CMC group, 5 for the WT–P80 group and 6 for the APC^min^–P80 group). Significance was determined using one-way group ANOVA with a Bonferroni test (* indicates statistical significance).

The increased number of adenomas observed in emulsifier-treated APC^min^ mice suggests the possibility of increased proliferation of intestinal epithelial cells. Hence, the proliferative status of colonic and small-intestine crypt cells was analyzed by Ki67 staining. We importantly observed that P80 consumption was sufficient to increase the number of Ki67-positive cells per crypt relative to the number in water-treated mice in both the colon and the small intestine of WT mice, while CMC increased crypts cells’ proliferation only in the colon of WT mice (Figure 3). Moreover, in genetically predisposed APC^min^ mice, CMC and P80 increased crypt cells’ proliferation in both the colon and the small intestine (Figure 3), demonstrating that the deleterious impact of emulsifiers on crypt cells proliferation is potentiated in the context of a genetical predisposition, a phenomenon that likely plays a role in the increased formation of intestinal adenomas.

### 2.3. Microinflammation Is Only Mildly Increased in the Intestinal Environment of Emulsifier-Treated APC^min^ Mice

**Figure 4:**
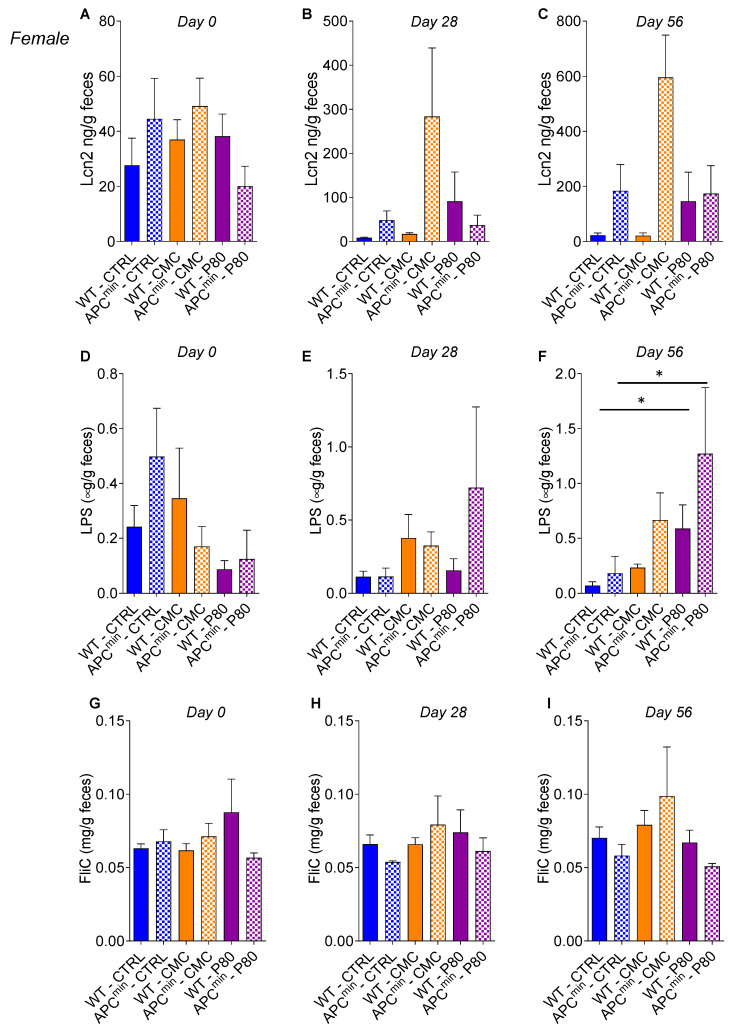
Dietary emulsifier consumption alters the intestinal inflammatory environment in WT and APC^min^ female mice. Seven-week-old WT and APC^min^ female mice were exposed to drinking water containing CMC or P80 (1.0%) for 15 weeks. (**A**–**C**) Fecal lipocalin-2 (Lcn2) concentration and (**D**–**F**) bioactive levels of fecal lipopolysaccharide (LPS) and flagellin (FliC) (**G**–**I**) were assessed at day 0 (**A**,**D**,**G**), day 28 (**B**,**E**,**H**), and day 56 (**C**,**F**,**I**). Data are the means +/− S.E.M. (*n* = 5 for the WT–CTRL group, 3 for the APC^min^–CTRL group, 7 for the WT–CMC group, 5 for the APC^min^–CMC group, 6 for the WT–P80 group and 3 for the APC^min^–P80 group). Significance was determined using one-way group ANOVA with a Bonferroni test (* indicates statistical significance).

**Figure 5:**
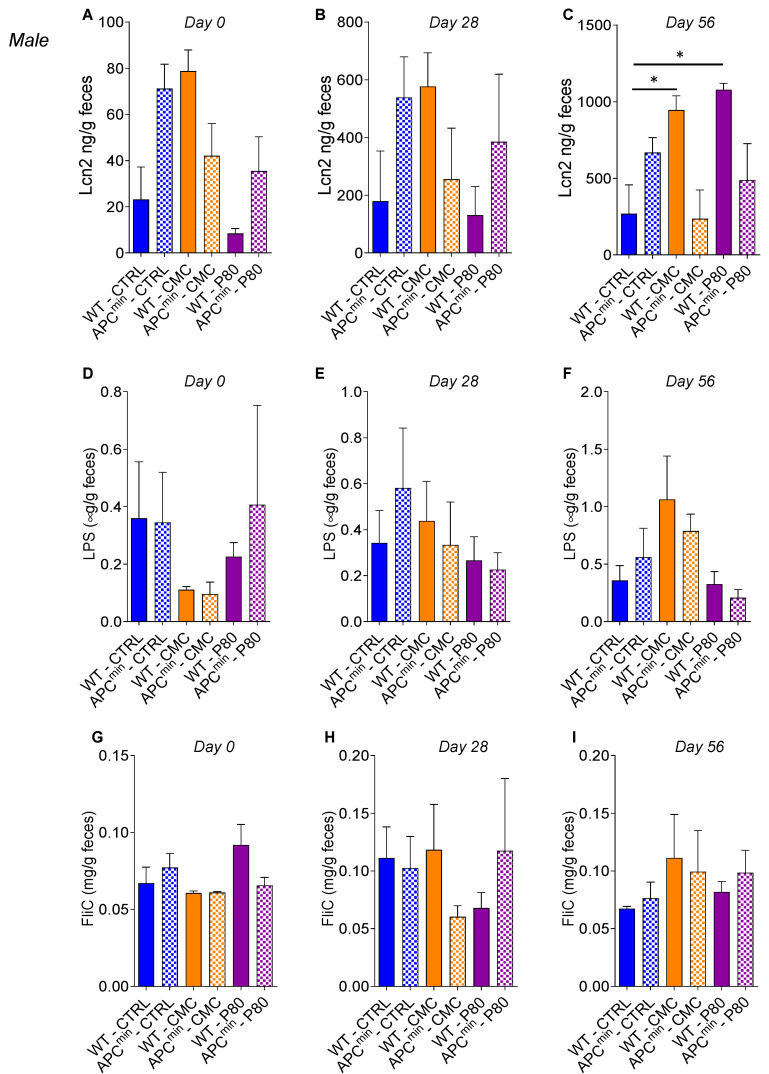
Dietary emulsifier consumption alters the intestinal inflammatory environment in WT and APC^min^ male mice. Seven-week-old WT and APC^min^ male mice were exposed to drinking water containing CMC or P80 (1.0%) for 15 weeks. (**A**–**C**) Fecal Lcn2 concentration and (**D**–**F**) bioactive levels of fecal LPS and FliC (**G**–**I**) were assessed at day 0 (**A**,**D**,**G**), day 28 (**B**,**E**,**H**), and day 56 (**C**,**F**,**I**). Data are the means +/− S.E.M. (*n* = 4 for the WT–CTRL group, 4 for the APC^min^–CTRL group, 3 for the WT–CMC group, 3 for the APC^min^–CMC group, 3 for the WT–P80 group and 5 for the APC^min^–P80 group). Significance was determined using one-way group ANOVA with a Bonferroni test (* indicates statistical significance).

Fecal lipocalin-2 (Lcn2), a sensitive and broadly dynamic marker of intestinal inflammation in mice [24], was used to quantify intestinal inflammation in emulsifier-treated WT and APC^min^ mice. In WT male mice, CMC and P80 consumption was associated with increased levels of fecal Lcn-2 at day 56 compared to non-treated WT mice (Figure 4A–C and Figure 5A–C). Moreover, in female mice, the APC^min^ genotype is associated with increased level of fecal Lcn2 compared to WT mice. Hence, these results suggest that the APC^min^ mutation or emulsifiers consumption alone are sufficient to induce some level of low-grade inflammation; interestingly, it seemed that there was no synergy between these two factors in emulsifier-treated APC^min^ mice, suggesting that emulsifiers consumption could aggravate intestinal adenomas formation through microinflammation-independent mechanisms. We next investigated the levels of bioactive fecal lipopolysaccharide (LPS) and flagellin (FLiC) in order to assess microbiota proneness to induce inflammation. In female mice, the levels of LPS, but not of FliC, were significantly increased in both WT and APC^min^ mice exposed to P80 (Figure 4D–I), while such effects were not observed in male mice (Figure 5D–I). Altogether, these results indicate that, contrary to what was described in a model of colitis-associated cancer [21], the increased number of intestinal polyps and the proliferative status observed in emulsifier-treated APC^min^ mice seem to be independent of an increase in the intestinal inflammatory tone.

### 2.4. Emulsifier Consumption Has Greater Impact on Microbiota Composition in APC^min^ Mice Compared with WT Mice

**Figure 6:**
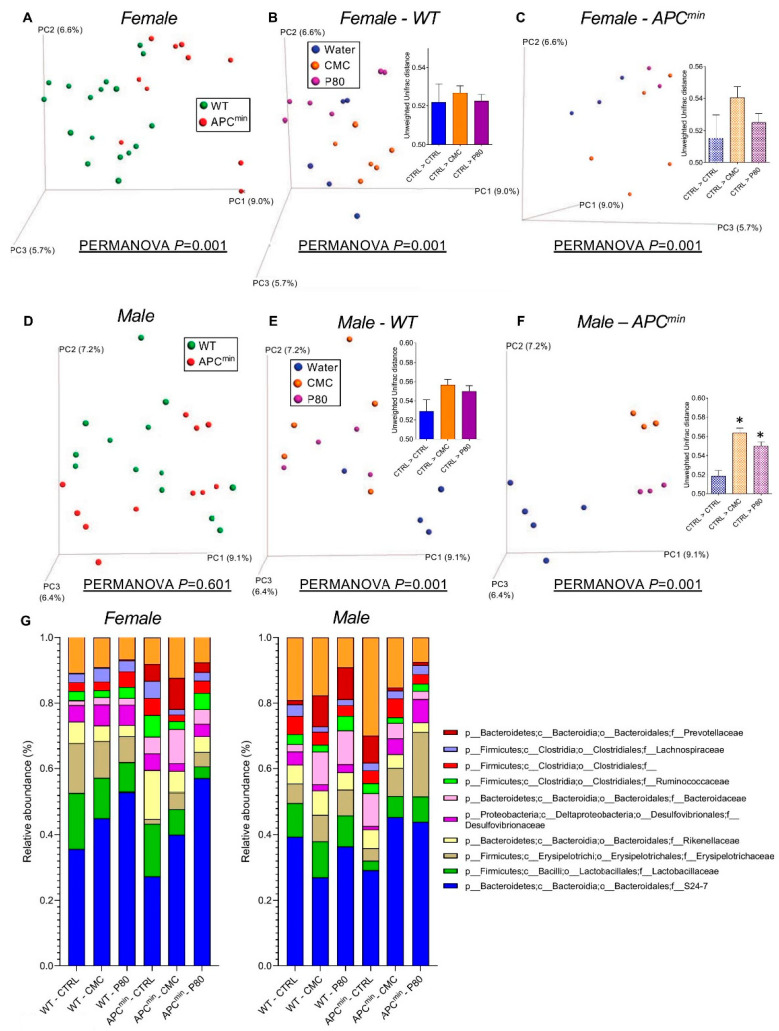
Dietary emulsifiers alter intestinal microbiota composition in WT and APC^min^ mice. Seven-week-old WT and APC^min^ mice were exposed to drinking water containing CMC or P80 (1.0%) for 15 weeks. Fecal microbiota composition was analyzed using Illumina sequencing of the V4 region of 16S rRNA genes. (**A**–**F**) Principal coordinate analysis of the unweighted UniFrac distance matrix of female (**A**–**C**) and male (**D**–**F**) mice. Histograms within the principal coordinates analysis (PCoA) plots represent the unweighted UniFrac distance separating water-treated animals from other groups (water, CMC, and P80). For cluster analysis on principal coordinate plots, categories were compared, and the statistical significance of clustering was determined using the Permanova method. (**G**) Taxonomic representation of the microbiota at the family level. For female mice, *n* = 5 for the WT–CTRL group, 3 for the APC^min^–CTRL group, 6 for the WT–CMC group, 5 for the APC^min^–CMC group, 6 for the WT–P80 group and 2 for the APC^min^–P80 group. For male mice, *n* = 4 for the WT–CTRL group, 5 for the APC^min^–CTRL group, 5 for the WT–CMC group, 3 for the APC^min^–CMC group, 4 for the WT–P80 group and 3 for the APC^min^–P80 group (* indicates statistical significance).

Fecal microbiota composition was analyzed by 16S Illumina sequencing of the 16S rRNA gene. We found that, as previously observed [26], the microbiota composition of APC^min^ mice differed from that of WT mice, in both female and male animals (Figure 6A,D). Moreover, in WT mice, emulsifier exposure led to alterations in microbiota composition, as previously reported [12] (Figure 6B,E). Interestingly, CMC and P80 consumption induced more drastic changes in microbiota composition in APC^min^ mice compared to WT mice (Figure 6B,C,E,F)**.** LEfSe (linear discriminant analysis (LDA) Effect Size) analysis, used to identify the most differentially abundant taxons between water- and emulsifier-treated groups, revealed a decrease in Actinobacteria upon emulsifier consumption in WT male and female mice, while some other microbiota alterations were observed in a sex-specific manner (Figure 6G and Figure S3). In APC^min^ mice, emulsifier consumption decreased the abundance of Clostridia, in both male and female mice, and increased the abundance of proteobacteria in male (Figure 6G and Figure S3). Altogether, these results demonstrate that CMC and P80 had more pronounced effects on the microbiota of the genetically susceptible mice APC^min^ compared to WT mice and could contribute to the observed increased number of intestinal polyps and enhanced proliferative status in emulsifier-treated APC^min^ mice.

## 3. Discussion

While a significant proportion of CRC are familial cancers attributable to inherited genetic mutations, a larger proportion is attributable to acquired genome modifications favored by risk factors such as smoking, alcohol consumption, obesity, physical inactivity, high consumption of red and processed meat, and low consumption of dietary fiber [27,28]. Mutations in the APC occur early in colorectal tumorigenesis and not only are responsible for familial adenomatous polyposis, but also play a role in the majority of non-inherited sporadic colorectal cancer [5]. Hence, genetics and environment act together in individuals with CRC predisposition, and there is a dire need to better understand these interactions.

Recent studies highlighted the existence of associations between consumption of ultra-processed food and higher risk of cancer [29]. Herein, we investigated if the consumption of dietary emulsifiers, commonly used food additives that we have previously shown to be sufficient to increase intestinal inflammation and tumor development in a mouse models of colitis-associated cancer [21], also impacts tumor development in a genetic model of CRC, namely, the APC^min^ mice model. We show in the present study that CMC- and P80-treated APC^min^ mice developed more tumors, which were also of larger size compared to those observed in water-treated control mice. This finding reinforces the concept that environment and genetics participate together in colon carcinogenesis and also suggests that dietary emulsifiers are dietary components involved in sporadic CRC.

Interestingly, the gut microbiota of APC^min^ mice is significantly altered in a way that can enhance tumor formation [30]. Mounting evidence undeniably suggests that the gut microbiota is an important determinant of CRC. A major tenet in this indictment is that microbial dysbiosis is a major driver of gut inflammation, which is strongly associated with an increased incidence of colon cancer [31]. In a recent study, the hypothesis that the gut microbiota from CRC patients promoted the progression of intestinal adenomas was tested by fecal transplant of CRC patients’ microbiota into APC^min^ mice [32]. A crucial role of the microbiota on the progression of intestinal adenomas was then established, in that the fecal transplant of CRC microbiota enhanced the progression of intestinal adenomas in APC^min^ mice [32]. In our previous study using a mouse model (AOM–DSS) of CAC, we observed that consumption of CMC and P80 resulted in altered gut microbiota composition and increased the levels of flagellin and LPS, indicating the presence of a low-grade pro-inflammatory environment [21]. In such CAC model, the proliferation/apoptosis balance was disturbed in a way that predisposed to aggravated tumor development [21]. Importantly, we also demonstrated that emulsifier-induced alterations in the microbiota were necessary and sufficient to drive alterations in intestinal epithelial cells’ homeostasis, with the observations that the effects of dietary emulsifiers were eliminated in mice devoid of microbiota (germ-free mice), while transplanting the microbiota from emulsifier-treated mice into germ-free mice was sufficient to induce alterations in the proliferation/apoptosis balance of intestinal epithelial cells [21]. Overall, this previous study revealed that the intestinal microinflammation caused by dietary emulsifiers consumption could promote colon carcinogenesis. A more recent study identified adherent-invasive *E. coli* (AIEC) as a microbiota member sufficient to trigger the detrimental effect of dietary emulsifiers on colonic carcinogenesis [33]. Importantly, it has also been shown that the intestinal microbiota composition can also influence tumor development by merely driving inflammation [10,34].

In the current study, we observed that the combination of both environmental (dietary emulsifier consumption) and genetic (APC^min^) factors exacerbate alterations in microbiota composition. A comparison of male and female cancer phenotypes revealed only a modest gender effect on cancer development following dietary emulsifier consumption. The impact of emulsifiers on the intestinal microbiota composition were only modest in WT mice, while APC^min^ mice showed significant alterations of their microbiota composition upon exposure to CMC and P80. The observation that the effects of dietary emulsifier exposure on intestinal inflammation and microbiota composition were only modest in WT mice align with our previous observation that the age of the mice at the beginning of emulsifier exposure matters, as more dramatic effects were observed when younger mice (three weeks of age) were exposed to emulsifiers [12]. Hence, these observations importantly suggest that dietary emulsifier consumption could lead to more drastic effects in APC^min^ mice if it starts early (around their weaning time). Moreover, even if we have previously reported the presence of detrimental effects of both CMC and P80 on metabolic health at doses as low as 0.1% [25], it will be important to analyze if this observation also applies to the promotion of tumor development observed in the APC^min^ mice model. With these limitations withstanding, this current study nonetheless importantly suggests that dietary emulsifiers are new CRC risk factors and that their effect on human intestinal physiology must be carefully evaluated through clinical trials.

## 4. Materials and Methods

### 4.1. Materials

Sodium CMC (average M_W_ ~ 250,000, degree of substitution = 0.7) and P80 were purchased from Sigma (Sigma, St. Louis, MO, USA).

### 4.2. Mice

Four-week-old male and female C57BL/6J WT and APC^min^ (Jackson Laboratory, stock number 002020) mice were used in this study. All mice were bred and housed at Georgia State University, Atlanta, Georgia, USA, under institutionally approved (25 September 2017) protocols (IACUC #A18006). Mice were housed in specific pathogen-free conditions and fed ad libitum with regular chow diet.

### 4.3. Emulsifier Agent Treatment

Seven-week-old mice were exposed to CMC or P80 diluted in drinking water (1.0%, Figure S1). The same water (reverse-osmosis-treated Atlanta city water) was used for the water-treated (control) group, and solutions were changed every week. Body weights were measured weekly and expressed as percentages of the initial body weight (day 0 defined as 100%) in order to study emulsifiers’ effect on body weight gain. Fresh feces were collected every week for subsequent analysis.

### 4.4. Tissue Collection

As schematized in Figure S1, after 15 weeks of emulsifier administration, blood was collected from the retrobulbar intraorbital capillary plexus. Hemolysis-free serum was generated by centrifugation of blood using serum separator tubes (Becton Dickinson, Franklin Lakes, NJ, USA). Mice were then euthanized, and colon length, colon weight, and spleen weight were measure. Organs and blood were collected for downstream analysis. Small intestinal and colonic tumors were counted, and their surface was measured. The total area of tumors for each colon was determined.

### 4.5. Ki67 Immunohistochemistry

Mouse ileum and proximal colon were fixed in 10% buffered formalin for 24 h at room temperature and subsequently embedded in paraffin. Tissues were sectioned at 8-µm thickness and deparaffinized. Antigen retrieval was performed by a 20 min incubation at 95 °C in sodium citrate buffer at pH 6. Immunohistochemistry was performed on an automated system (Bond III Leica), with a 20 min incubation with anti-Ki67 (1:500, Abcam, ab15580, Cambridge, UK) at 37 °C. Staining was revealed with the Bond Polymer Refine Detection kit (Leica, DS9800, Wetzlar, Germany), which includes Mayer’s hematoxylin counterstaining of the nuclei. After dehydration, the slides were covered with a coverslip and scanned with a Lamina scanner from Perkin Elmer. Ki67-positive cells were counted per crypt.

### 4.6. Quantification of Fecal Lcn-2 by ELISA

For quantification of fecal Lcn-2 by ELISA, frozen fecal samples were reconstituted in PBS containing 0.1% Tween 20 to a final concentration of 100 mg/mL and vortexed for 20 min to get a homogenous fecal suspension [35]. These samples were then centrifuged, and supernatants were collected and used for estimating Lcn-2 levels using the Duoset murine Lcn-2 ELISA kit (R&D Systems, Minneapolis, MN, USA).

### 4.7. Fecal Flagellin and Lipopolysaccharide Load Quantification

We quantified flagellin and LPS as previously described [36] using human embryonic kidney (HEK)-Blue-mTLR5 and HEK-BluemTLR4 cells, respectively (Invivogen, San Diego, CA, USA). We resuspended fecal material in PBS to a final concentration of 100 mg/mL and homogenized it using a Mini-Beadbeater-24 without the addition of beads to avoid bacteria disruption. Supernatants were serially diluted and applied to mammalian cells. Purified *E. coli* flagellin and LPS (Sigma, St Louis, MI, USA) were used for standard-curve determination. After 24 h of stimulation, we applied cell culture supernatants to QUANTI-Blue medium (Invivogen, San Diego, CA, USA) and measured alkaline phosphatase activity at 620 nm after 30 min.

### 4.8. Fecal Microbiota Analysis by 16S rRNA Gene Sequencing Using Illumina Technology

We performed 16S rRNA gene amplification and sequencing using the Illumina MiSeq technology following the protocol of Earth Microbiome Project with modifications (www.earthmicrobiome.org/emp-standard-protocols accessed on 1 December 2020) [37,38]. Bulk DNA was extracted from frozen feces using a Qiagen Power Fecal DNA Isolation Kit with mechanical disruption (bead beating). The 16S rRNA genes, region V4, were PCR-amplified from each sample using a composite forward primer and a reverse primer containing a unique 12-base barcode, designed using the Golay error-correcting scheme, which was used to tag the PCR products from their respective samples [38]. We used the forward primer 515F 5′- *AATGATACGGCGACCACCGAGATCTACACGCT*XXXXXXXXXXXXTATGGTAATT*GT*GTGYCAGCMGCCGCGGTAA-3′: the italicized sequence is the 5′ Illumina adapter, the 12× sequence is the golay barcode, the bold sequence is the primer pad, the italicized and bold sequence is the primer linker, and the underlined sequence is the conserved bacterial primer 515F. The reverse primer 806R used was 5′-*CAAGCAGAAGACGGCATACGAGAT*AGTCAGCCAG*CC*GGACTACNVGGGTWTCTAAT-3′: the italicized sequence is the 3′ reverse complement sequence of Illumina adapter, the bold sequence is the primer pad, the italicized and bold sequence is the primer linker, and the underlined sequence is the conserved bacterial primer 806R. PCR reactions consisted of Hot Master PCR mix (Quantabio, Beverly, MA, USA), 0.2 µM of each primer, and 10–100 ng of template, and the reaction conditions were 3 min at 95 °C, followed by 30 cycles of 45 s at 95 °C, 60s at 50 °C, and 90 s at 72 °C on a Biorad thermocycler. The PCR products were purified with Ampure magnetic purification beads (Agencourt, Brea, CA, USA) and visualized by gel electrophoresis. The products were then quantified (BIOTEK Fluorescence Spectrophotometer, Winooski, VT, USA) using the Quant-iT PicoGreen dsDNA assay. A master DNA pool was generated from the purified products in equimolar ratios. The pooled products were quantified using the Quant-iT PicoGreen dsDNA assay and then sequenced using an Illumina MiSeq sequencer (paired-end reads, 2 × 250 bp) at Cornell University, Ithaca, NY, USA.

### 4.9. Gene Sequence Analysis of 16S rRNA

Forward and reverse Illumina reads were joined using the fastq-join method [39,40], and sequences were demultiplexed and quality-filtered using the Quantitative Insights Into Microbial Ecology (QIIME, version 1.8.0) software package [41]. QIIME default parameters were used for quality filtering (reads truncated at first low-quality base and excluded if (1) there were more than three consecutive low-quality base calls (2), less than 75% of read length was consecutive high-quality base calls (3), at least one uncalled base was present (4), more than 1.5 errors were present in the bar code (5), any Phred qualities were below 20, or (6) the length was less than 75 bases. Sequences were assigned to operational taxonomic units (OTUs) using the UCLUST algorithm [42] with a 97% threshold of pairwise identity (with the creation of new clusters with sequences that did not match the reference sequences) and classified taxonomically using the Greengenes reference database 13_8 [43]. A single representative sequence for each OTU was aligned, and a phylogenetic tree was built using FastTree [44]. The phylogenetic tree was used for computing the unweighted UniFrac distances between samples [45,46], and rarefaction was performed and used to compare abundances of OTUs across samples. PCoA plots were used to assess the variation between the experimental groups (beta diversity). Unprocessed sequencing data were deposited in the Genome Sequence Archive (GSA) in BIG Data Center, Beijing Institute of Genomics, Chinese Academy of Sciences, under accession number CRA005280, publicly accessible at https://bigd.big.ac.cn/gsa accessed on 1 December 2020.

### 4.10. Statistical Analysis

Data are presented as means ± SEM. Significance was determined by one-way group ANOVA with Bonferroni’s multiple comparisons test (GraphPad Prism software, version 8); * indicates statistically significant differences.

## Data Availability

Unprocessed sequencing data were deposited in the Genome Sequence Archive (GSA) in BIG Data Center, Beijing Institute of Genomics, Chinese Academy of Sciences, under accession number CRA005278, publicly accessible at https://bigd.big.ac.cn/gsa accessed on 1 December 2020.

## Supplementary Materials

The following are available online at https://www.mdpi.com/1422-0067/22/5/2602/s1, Figure S1, Figure S2, Figure S3.

## Abbreviations

APC	adenomatosis polyposis coli
AOM	azoxymethane
CMC	carboxymethylcellulose
DSS	Dextran sulfate sodium
IBD	inflammatory bowel disease
Lcn-2	lipocalin-2
LEfSe	LDA Effect Size
LPS	lipopolysaccharide
Min	multiple intestinal neoplasia
P80	polysorbate-80
SI	small intestine
TLR4	Toll-like receptor 4
TLR5	Toll-like receptor 5
