# Supplementary material for: Consumption of Select Dietary Emulsifiers Exacerbates the Development of Spontaneous Intestinal Adenoma
Source: Int J Mol Sci. 2021 Mar 5;22(5):2602. doi: 10.3390/ijms22052602 (PMC7961571; doi:10.3390/ijms22052602)
Supplement: Supplementary file 1 [file ijms-22-02602-s001.zip › ijms-1107941-supplementary.pptx]

## Slide 1
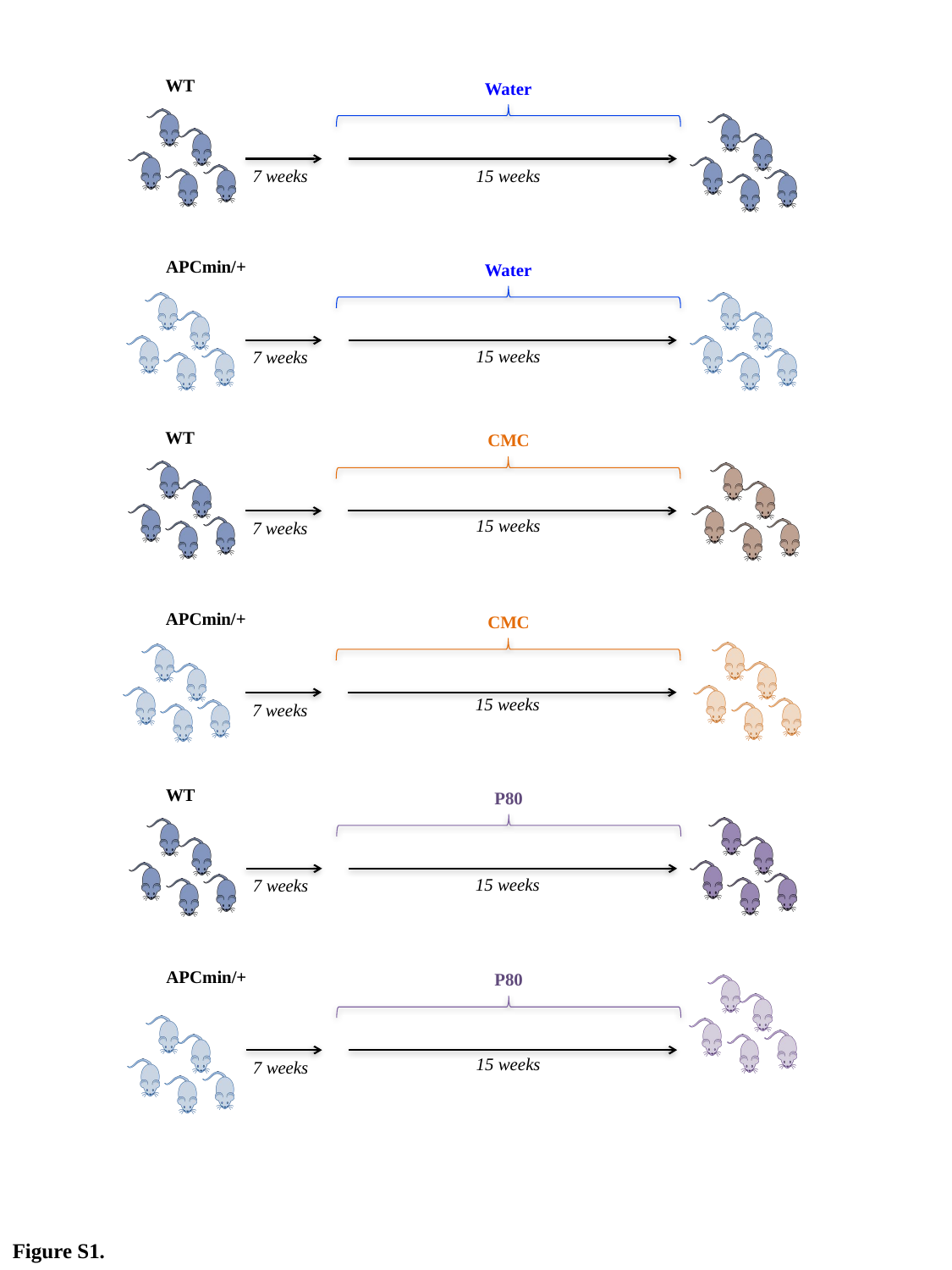

WT
Water
7 weeks
15 weeks
APCmin/+
Water
15 weeks
7 weeks
WT
CMC
15 weeks
7 weeks
APCmin/+
CMC
15 weeks
7 weeks
WT
P80
15 weeks
7 weeks
APCmin/+
P80
15 weeks
7 weeks
Figure S1.

## Slide 2
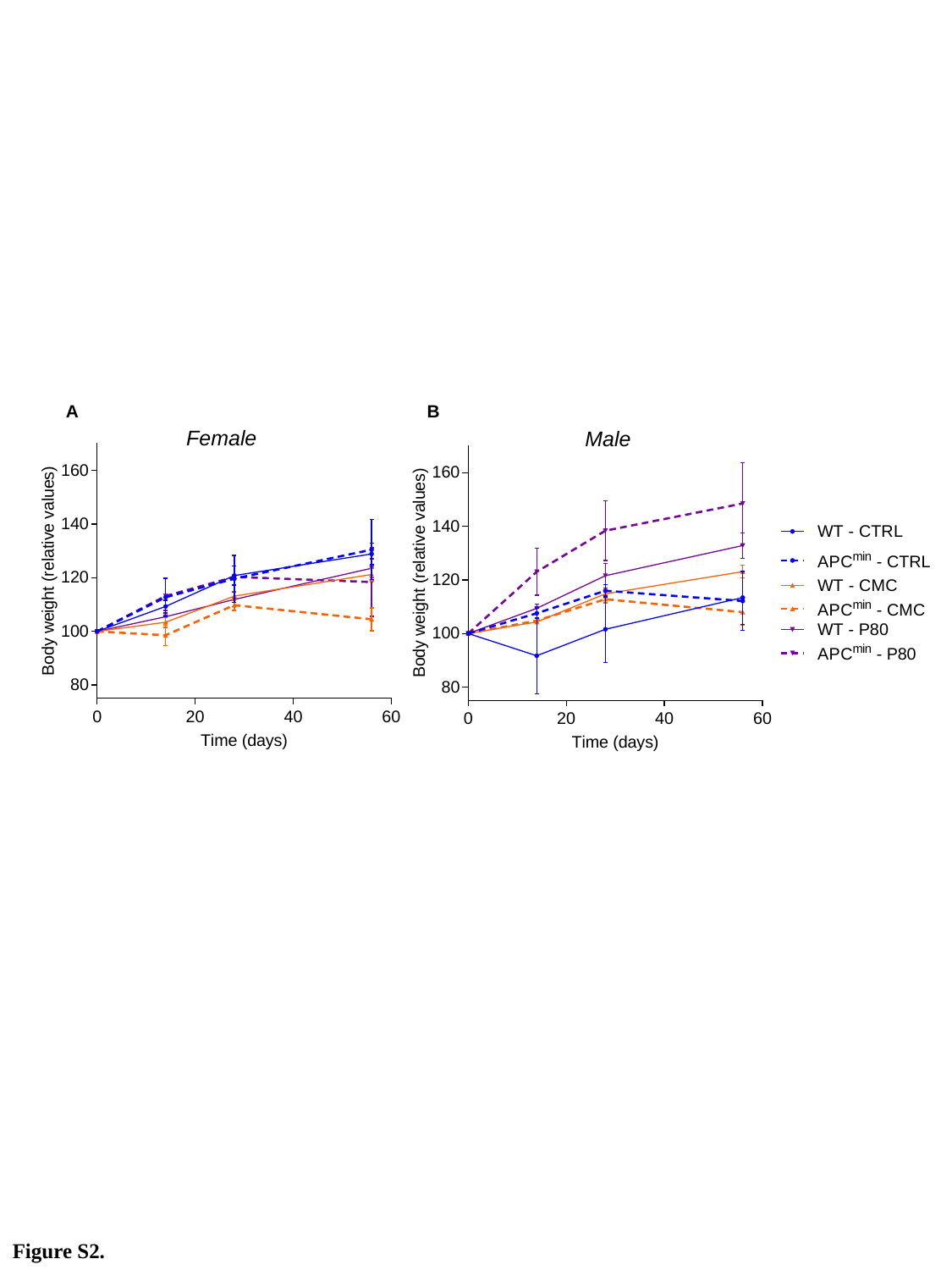

A
B
Female
Male
Figure S2.

## Slide 3
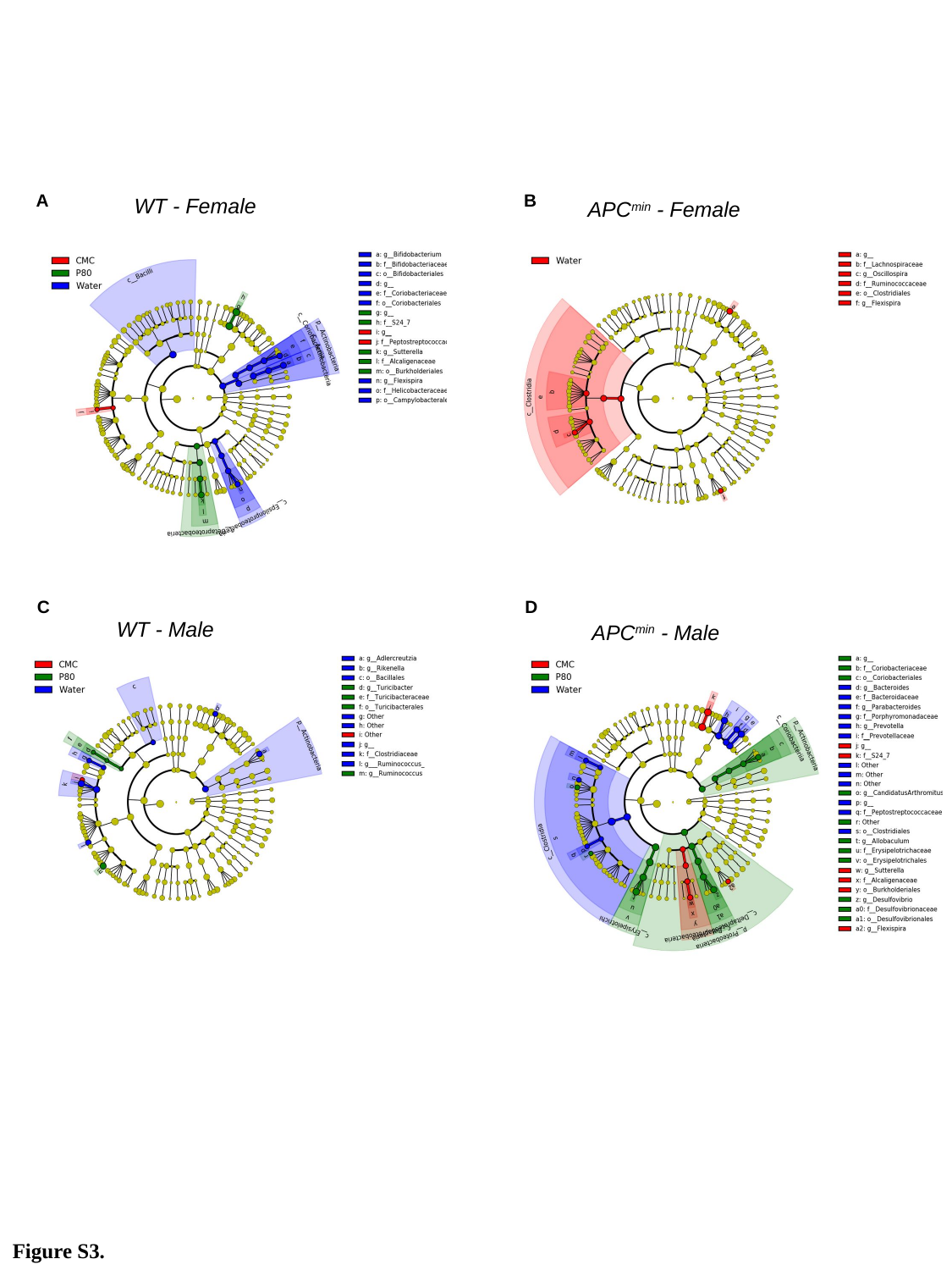

A
B
WT - Female
APCmin - Female
C
D
WT - Male
APCmin - Male
Figure S3.
